# Patterned Lead‐Free Double Perovskite/Polymer Fluorescent Piezoelectric Composite Films for Advanced Anti‐Counterfeiting

**DOI:** 10.1002/advs.202409692

**Published:** 2024-11-11

**Authors:** Jindou Shi, Zeyu Wang, Nanxiang Jia, Minqiang Wang, Youlong Xu, Xiangming Li, Jinyou Shao

**Affiliations:** ^1^ Electronic Materials Research Laboratory Key Laboratory of the Ministry of Education International Center for Dielectric Research&Shaanxi Engineering Research Center of Advanced Energy Materials and Devices Xi'an Jiaotong University Xi'an 710049 China; ^2^ Frontier Institute of Science and Technology (FIST) Xi'an Jiaotong University Xi'an 710049 China; ^3^ Micro‐ and Nano‐technology Research Center of State Key Laboratory for Manufacturing Systems Engineering Xi'an Jiaotong University Xi'an 710049 China

**Keywords:** anti‐counterfeiting, Cs_2_Na_0.8_Ag_0.2_BiCl_6_:Yb^3+^/Er^3+^/P(VDF‐TrFE), fluorescent, patterned, piezoelectric

## Abstract

The limitations of single fluorescent anti‐counterfeiting technologies necessitate the development of more sophisticated encryption methods to protect information and data. Traditional optical anti‐counterfeiting encryption techniques, which rely on light sources with varying wavelengths to identify information, are now insufficient to meet contemporary security demands due to their restricted response to a narrow range of wavelengths. In this study, the fabrication of patterned, lead‐free double perovskite (DP)/poly(vinylidene fluoride‐trifluoroethylene) (P(VDF‐TrFE)) fluorescent piezoelectric composite films (CFs) is reported. These CFs integrate the up‐conversion and down‐conversion photoluminescent properties of Cs_2_Na_0.8_Ag_0.2_BiCl_6_:Yb^3+^/Er^3+^ DP crystals with the piezoelectric properties of P(VDF‐TrFE) film, facilitating multi‐modal information protection. The fluorescent signals of different concealed information in CFs are observable under the excitation of 365 nm UV light and 980 nm infrared (IR) light. Additionally, external pressure applied at various locations on the CFs generates corresponding electrical signals, thereby providing triple‐layer encryption for protected information. A multifunctional anti‐counterfeiting device has been further developed by integrating patterned optical and electrical responses onto flexible CFs, achieving synergistic protection of information security in cross fields and bringing a significant advancement to the high‐level anti‐counterfeiting market.

## Graphical abstract

A flexible multifunctional anti‐counterfeiting device has been developed by integrating patterned multi‐modal fluorescent anti‐counterfeiting technology with piezoelectric anti‐counterfeiting technology into a flexible organic thin film, achieving synergistic protection of information security in cross fields, contributing to further upgrade in anti‐counterfeiting level.

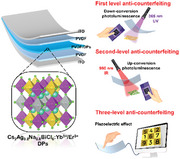

## 1 Introduction

In recent years, numerous advanced anti‐counterfeiting technologies have been developed for information encryption as information security standards have continuously evolved.^[^ ^1^, ^2^, ^3^, ^4^, ^5^, ^6^, ^7^, ^8^, ^9^ ^]^ Among these, fluorescent anti‐counterfeiting technology has been particularly favored due to its adjustable optical emission and strong fluorescent intensity,^[^ ^10^, ^11^, ^12^, ^13^, ^14^ ^]^ which make it easily identifiable in practical applications and thus provide a higher level of security for protected information.^[^ ^15^, ^16^, ^17^ ^]^ However, as manufacturing processes have advanced, counterfeiting technology has also rapidly developed,^[^ ^18^, ^19^ ^]^ rendering traditional mono‐modal fluorescent anti‐counterfeiting insufficient to meet the encryption requirements for information security in the anti‐counterfeiting market.^[^ ^20^, ^21^, ^22^, ^23^ ^]^ Therefore, enhancing information security by integrating multi‐modal anti‐counterfeiting technologies for deeper encryption will become the mainstream approach in subsequent anti‐counterfeiting developments.

Currently, many researchers have employed multi‐modal fluorescence emission for anti‐counterfeiting encryption, achieving information decryption under various excitation light sources and environments by utilizing multiple fluorescence‐dependent emissions.^[^ ^24^, ^25^, ^26^, ^27^, ^28^, ^29^, ^30^, ^31^ ^]^ Ding et al. observed both efficient and tunable dual‐modal fluorescence emission under 254 nm ultraviolet (UV) and 980 nm infrared (IR) laser excitation through the doping of rare‐earth ions, designing encrypted QR codes to ensure information security.^[^ ^32^ ^]^ Zhou et al. developed multi‐modal fluorescent anti‐counterfeiting labels consisting of NaErF_4_‐cored core–multishell up‐conversion (UC) nanoparticles (NPs) by modulating several structural factors, displaying red, green, and blue luminescence under 808, 980, and 1550 nm excitation, respectively.^[^ ^33^ ^]^ Shi et al. enhanced the level of information security by incorporating Yb^3+^ and Er^3+^ into lead‐free double perovskite (DP) material, which not only exhibited multi‐modal fluorescence emission under different excitation sources, but also showed tunable fluorescence intensity with varying environmental temperatures.^[^ ^34^ ^]^ Obviously, these anti‐counterfeiting techniques primarily rely on the fluorescent properties of materials, which are too monolithic for some advanced anti‐counterfeiting applications. Thus, the integration of multi‐disciplinary anti‐counterfeiting techniques for deep encryption is necessary to further ensure the foolproof of information.

**Figure 1:**
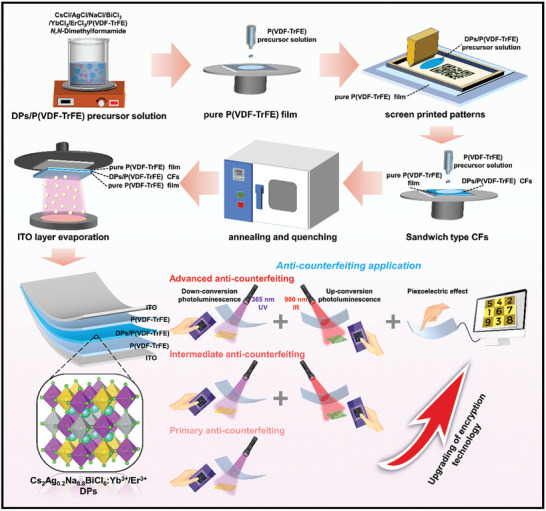
Schematic illustration of the preparation process for flexibly patterned fluorescent piezoelectric Cs_2_Na_0.8_Ag_0.2_BiCl_6_:Yb^3+^/Er^3+^/P(VDF‐TrFE) CFs, and its conceptual demonstration in multimodal anti‐counterfeiting applications.

Here, we refer to the optimal doping ratio of rare earth ions (8% Yb^3+^ and 2% Er^3+^) in our previous work,^[^ ^34^ ^]^ and choose a new DP (Cs_2_Na_0.8_Ag_0.2_BiCl_6_) as the host to obtain Cs_2_Na_0.8_Ag_0.2_BiCl_6_:Yb^3+^/Er^3+^ DP crystals with multimode fluorescence emission. Subsequently, a patterned fluorescent piezoelectric film was developed by integrating the multimode fluorescence emission of the Cs_2_Na_0.8_Ag_0.2_BiCl_6_:Yb^3+^/Er^3+^ DP crystals with a piezoelectric generator made from poly(vinylidene fluoride‐trifluoroethylene) (P(VDF‐TrFE)). Patterned fluorescent piezoelectric Cs_2_Na_0.8_Ag_0.2_BiCl_6_:Yb^3+^/Er^3+^/P(VDF‐TrFE) composite films (CFs) with high brightness and fast responsiveness were fabricated through patterned printing, annealing, and the introducing ITO capping layers, followed by film polarization control at high voltage. These Cs_2_Na_0.8_Ag_0.2_BiCl_6_:Yb^3+^/Er^3+^/P(VDF‐TrFE) CFs exhibited different hidden information under 365 nm UV and 980 nm IR irradiation, enabling the dual‐modal fluorescence encryption. Subsequently, the piezoelectric output generated by pressing was decoded by the signal recognition terminal to obtain the tertiary cryptographic information (**Figure** **1**). In conclusion, a flexible multifunctional anti‐counterfeiting device has been developed by integrating patterned multi‐modal fluorescent anti‐counterfeiting technology with piezoelectric anti‐counterfeiting technology into a flexible organic thin film. This innovation realizes synergistic protection of information security in cross fields, contributing to further upgrade in anti‐counterfeiting level.

## 2 Results and Discussion

**Figure 2:**
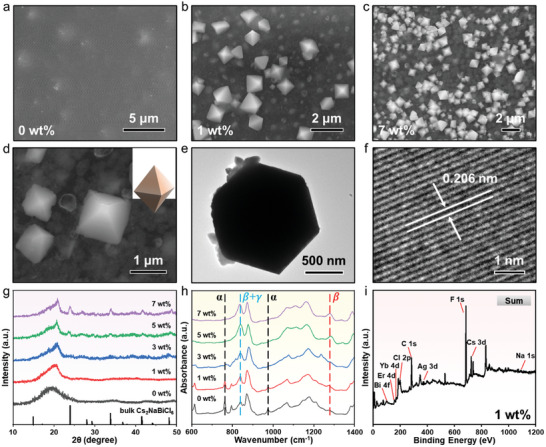
SEM images of a) pure P(VDF‑TrFE) film, b) 1 wt.% DP/P(VDF‑TrFE) CFs, c) 7 wt.% DP/P(VDF‑TrFE) CFs and d) magnified 7 wt.% DP/P(VDF‑TrFE) CFs. e) TEM and f) High resolution‐TEM images of DP crystal. g) XRD patterns of CFs with varying DP content, the bottom corresponds to a pure bulk Cs_2_NaBiCl_6_ (ICSD: 2738). h) FTIR spectra of CFs with varying DP content. i) XPS spectrum of 1 wt.% DP/P(VDF‑TrFE) CFs.

The microscopic morphology and structure of CFs with varying DP content obtained by patterning were further investigated. Compared to pure P(VDF‑TRFE) films obtained under the same conditions (**Figure** **2**a), many distinct crystals were embedded in CFs with an average size of ≈900 nm (Figure 2b,c; Figure S1 and S2, Supporting Information), suggesting that Cs_2_Na_0.8_Ag_0.2_BiCl_6_:Yb^3+^/Er^3+^ DP crystals would be in situ grown in P(VDF‑TrFE) after annealing and quenching. Meanwhile, the proportion of precursors directly controlled the distribution of DP crystals in CFs, and higher proportion inputs of chloride salts resulted in the more densely distributed Cs_2_Na_0.8_Ag_0.2_BiCl_6_:Yb^3+^/Er^3+^ crystals in the same size P(VDF‑TrFE) films (Figure 2b,c; Figure S1a,b, Supporting Information). Subsequently, the magnified scanning electron microscope (SEM) image displayed that DP crystals in CFs presented an octahedral structure, and the surface was smooth with no other products being observed (Figure 2d), which was consistent with the previous report of Cs_2_NaBiCl_6_/Mn^2+^ crystals,^[^ ^35^ ^]^ reconfirming that the patterning method was able to achieve in situ doping of Ag^+^, Yb^3+^, and Er^3+^ in Cs_2_NaBiCl_6_ crystals, laying the foundation for the regulation of its fluorescence properties. Furthermore, the transmission electron microscope (TEM) image indicated that Cs_2_Na_0.8_Ag_0.2_BiCl_6_:Yb^3+^/Er^3+^ crystals in CFs were highly crystallized (Figure 2e), with the surface lattice spacing of 0.206 nm (Figure 2f), which benefited from the annealing and quenching process that supported the growth of DPs. Especially, X‐ray diffraction (XRD) was employed to analyze the crystal structure of DP/P(VDF‑TrFE) CFs. As the DP content in CFs increased, the diffraction peaks of Cs_2_Na_0.8_Ag_0.2_BiCl_6_:Yb^3+^/Er^3+^ crystals were gradually observed in the XRD patterns (Figure 2g), which were slightly shifted compared to the reference Cs_2_NaBiCl_6_ at the bottom, resulting mainly from the lattice vibration triggered by ion doping,^[^ ^36^, ^37^ ^]^ and confirming the crystalline growth of DP crystals in P(VDF‑TrFE) films. When the content of DP reached 7 wt.%, the characteristic diffraction peaks of DP appeared in DP/P(VDF‐TrFE) CFs, indicating that the high content of DP would migrate to the surface of the film and be exposed to the environment.^[^ ^38^ ^]^ Interestingly, according to the different arrangements of the polymer chains, the diffraction peaks at 18.4°, 20.2°, and 20.8° of the pure P(VDF‑TrFE) film correspond to its α‐, γ‐, and β‐phases, respectively.^[^ ^39^ ^]^ Of these, the polar β‐phase and semipolar γ‐phase mainly supported the piezoelectric properties of P(VDF‑TrFE) film, while the nonpolar α‐phase did not contribute, so enhancing the piezoelectric output of the film by improving the ratio of the β‐phase and γ‐phase has been mandatory.^[^ ^38^, ^40^ ^]^ Meanwhile, some vibrational peaks in the Fourier transform infrared (FTIR) spectra of DP/P(VDF‐TrFE) CFs represented different electroactive phases, including the non‐polar α‐phase (765 and 975 cm^−1^), the polar β‐phase (1275 cm^−1^), the semipolar γ‐phase (1234 cm^−1^), and the overlapping β‐ and γ‐phases (840 cm^−1^) (Figure 2h ).^[^ ^38^ ^]^ To quantitatively compared the variation in the electroactive phase ratios of the P(VDF‑TrFE) film with different DP content, the percentage of the respective phases was calculated by using peak fitting (Table S1, Supporting Information).^[^ ^41^, ^42^ ^]^ Due to the ionic nature of Cs_2_Na_0.8_Ag_0.2_BiCl_6_:Yb^3+^/Er^3+^ crystals that allowed the production of negatively charged ions to interact with the positively charged CH_2_ group in P(VDF‑TrFE) films, thereby inducing the formation of the more polar β‐phase.^[^ ^41^ ^]^ Calculations showed that high electroactive phase ratio can be achieved when the doping ratio of DP was 5 wt.%, with 48% for the β‐phase and 35% for the γ‐phase (Table S1). It was attributed to the fact that the lower DP contents were not sufficient to fully polarize P(VDF‐TrFE) film, but the agglomeration of DP crystals on the polymer surface at ultra‐high content would expose the ionic semiconductor DP to the environment, accelerating the surface charge dissipation, thereby hindering the generation of the β‐phases,^[^ ^38^, ^41^, ^43^ ^]^ and degrading the electrical properties, which was confirmed by the XRD results. Subsequently, the surface morphology of DP/P(VDF‑TrFE) CFs with different DP content in a 10×10 µm^2^ area was obtained by using an atomic force microscope (AFM) (Figure S3, Supporting Information). It can be seen that the surface of the pure P(VDF‑TrFE) film was uniform, flat, and smooth, and the surface roughness R_q_ (root mean square roughness) and R_a_ (arithmetical mean deviation of the profile) were 5.3 and 3.06 nm, respectively (Figure S3a, Supporting Information). However, the surface roughness of CFs was elevated with the increasing of DP content, and the R_q_ and R_a_ of 7 wt.% DP/P(VDF‑TrFE) CFs reached 98.5 and 74.2 nm, respectively (Figure S3e, Supporting Information), which mainly stemmed from the aggregation of DP inside the CFs to the surface location of the film, inducing a change in the surface roughness of the CFs. The roughness variation of DP/P(VDF‑TrFE) CFs supported the results of XRD patterns and FTIR spectra, confirming the close correlation between the electroactive phase in CFs and the DP content. Therefore, the composite of DP crystals in moderate amounts can effectively promote the crystallization of β‐phase in P(VDF‑TrFE) films, thereby ensuring that DP/P(VDF‑TrFE) CFs maintained excellent electrical properties. Finally, the elemental composition in 1 wt.% DP/P(VDF‑TrFE) CFs, with the minimum DP content, was analyzed using X‐ray photoelectron spectroscopy (XPS), but the signal of Cs_2_Na_0.8_Ag_0.2_BiCl_6_:Yb^3+^/Er^3+^ crystals could not be detected at the surface of CFs. After 1100 s etching of the ion beam, all elements were probed in the interior of DP/P(VDF‑TrFE) CFs (Figure 2i), suggesting that the lower content DPs were concentrated in the middle of CFs, which will avoid the dissipation of the surface charge, contributing to the enhancement of the electrical properties. The corresponding high‐resolution spectra of the elements showed slight displaced positions of all peaks (Figure S4a–i, Supporting Information), which can be attributed to the increased electron binding energy of the elements caused by the formation of Cs_2_Na_0.8_Ag_0.2_BiCl_6_:Yb^3+^/Er^3+^ crystals, and reflecting again that the in situ growth of DPs can be achieved while the patterned CFs has been annealed and quenched. Subsequently, all the constituent elements in 1 wt.% DP/P(VDF‑TrFE) CFs were also detected in the corresponding energy dispersive X‐ray spectra (EDS) (Figure S5, Supporting Information), which were consistent with the results acquired by XPS, confirming the completion of the composite between Cs_2_Na_0.8_Ag_0.2_BiCl_6_:Yb^3+^/Er^3+^ crystals and P(VDF‐TrFE) films. In addition, all elements were detected in the XPS spectrum of the 7 wt.% DP/P(VDF‑TrFE) CFs (Figure S6a, Supporting Information), and the high‐resolution XPS spectra of each element showed no significant changes in the characteristic peaks (Figure S6b–j, Supporting Information), which was consistent with the results of the XPS spectra of the 1 wt.% DP/P(VDF‑TrFE) CFs. However, all elemental signals were detected on the surface of 7 wt.% DP/P(VDF‑TrFE) CFs without undergoing ion beam etching during testing. This phenomenon reconfirmed that some DP would migrate to the surface of CFs with increasing DP content, thereby influencing their electrical properties. Combined above characterization results indicated that the CFs with varying DP content obtained by patterning not only in situ grown Cs_2_Na_0.8_Ag_0.2_BiCl_6_:Yb^3+^/Er^3+^ crystals but also caused the proportion of polar β‐phase in P(VDF‑TrFE) films to be elevated, which will make it promising in the field of optical and electrical applications.

**Figure 3:**
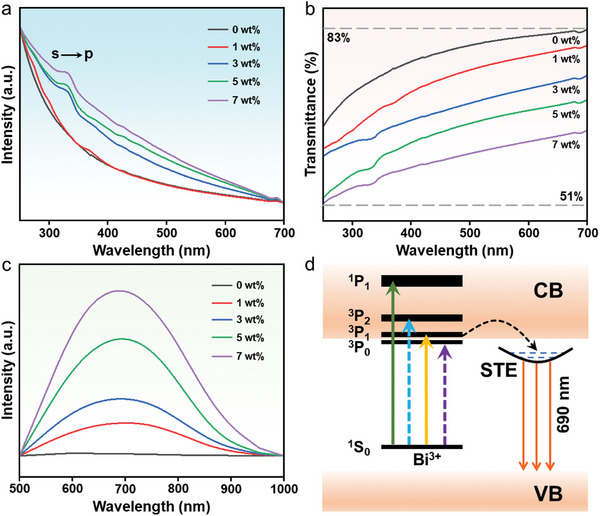
a) Absorbance, b) transmittance, and c) DC (365 nm excitation) PL spectra of CFs with varying DP content. d) Energy level diagram and DC PL mechanism of Cs_2_Na_0.8_Ag_0.2_BiCl_6_:Yb^3+^/Er^3+^. CB and VB represent the conduction and valence bands of the host, respectively.

**Figure 4:**
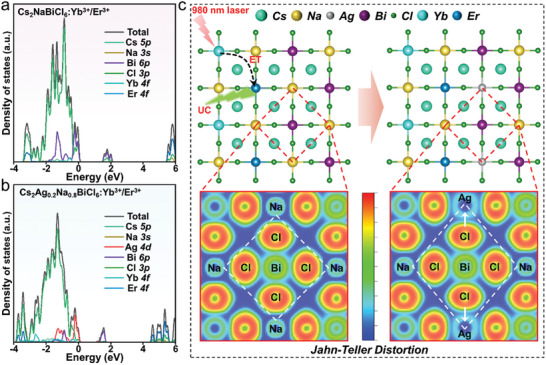
Projected density of states (PDOS) for a) Cs_2_NaBiCl_6_:Yb^3+^/Er^3+^ and b) Cs_2_Na_0.8_Ag_0.2_BiCl_6_:Yb^3+^/Er^3+^. c) The electron localization function change of Cs_2_NaBiCl_6_:Yb^3+^/Er^3+^ after Ag^+^ ion replaced Na^+^ ion, and schematic diagram of Jahn‐Teller distortion caused by Ag^+^ doping.

To further assess the prospect of CFs with varying DP content for multimodal fluorescence anti‐counterfeiting applications, the down‐conversion (DC) fluorescence properties of CFs were measured by a series of optical characterizations. The UV–vis absorbance spectra of CFs with low DP content (1 wt.%) almost overlapped with the undoped film (0 wt.%) due to the masking of surface P(VDF‐TrFE) film (**Figure** **3**a). However, an exciton absorption peak located at 320 nm was gradually observed in the absorbance spectra of CFs with the increasing content of DPs, which originated from the s→p orbital transition of Bi^3+^ in DPs, suggesting that CFs can effectively absorb UV light energy.^[^ ^35^, ^44^ ^]^ In order to avoid the masking of the surface P(VDF‐TrFE) film, pure Cs_2_Na_0.8_Ag_0.2_BiCl_6_:Yb^3+^/Er^3+^ DP powder was prepared by solution method. By analyzing the absorbance spectrum (Figure S7, Supporting Information), it contained three absorption bands, all related to the 6s^2^→6s^1^p^1^ transition of the [BiCl_6_]^3−^octahedron (Figure S7a, Supporting Information). Among them, the broad peak at 270 nm was designated as a spin forbidden ^1^S_0_→^3^P_2_ transition, while the peak at 320 nm was designated as a partially allowed ^1^S_0_→^3^P_1_ transition, and the shoulder peak at 375 nm was designated as a forbidden ^1^S_0_→^3^P_0_ transition. Moreover, the spin‐allowed ^1^S_0_→^1^P_1_ transition has been reported to be located ≈200 nm, a region beyond the detection range of our instruments.^[^ ^45^ ^]^ Owing to the masking of the surface P(VDF‐TrFE) film (Figure S7b, Supporting Information), it allowed only the ^1^S_0_→^3^P_1_ transition located at 320 nm to be observed in the absorbance spectrum of the DP/P(VDF‑TrFE) CFs (Figure 3a). At the same time, the transmittance corresponding to CFs gradually decayed to 51% (Figure 3b), which was attributed to the enhancement of DPs content, implying that the DPs content in the patterned CFs can be controlled by adjusting the ratio of the initial precursors. Subsequently, the DC fluorescence properties of CFs with varying DP content were explored under the excitation of 365 nm UV light (Figure 3c). A broad orange fluorescence emission peak at 690 nm was observed in the PL spectra of CFs with varying DP content, corresponding to the color coordinates in the International Commission on Illumination (CIE) diagram at (0.56, 0.44) (Figure S8, Supporting Information), which originated from the self‐trapping exciton (STE) emission of DP in CFs. Subsequently, the DC emission mechanism of CFs was simulated, benefiting from the alloying of Ag^+^ and Na^+^, the forbidden transition of Bi^3+^ was broken, thereby enabling more effective energy transfer to the STE states, emitting a bright orange light at 690 nm (Figure 4d). Moreover, the PL intensity of CFs was gradually enhanced with the increase of DP content (Figure 3c), and the DC photoluminescence quantum yield (PLQY) of 7 wt.% DP/P(VDF‐TrFE) CFs with the most outstanding optical performance was 27.64% (Figure S9a, Supporting Information), which laid the foundation for its subsequent application in the field of optical anti‐counterfeiting.

Up to now, alloying has been regarded as a convenient way to optimize the optical properties of DPs, taking Cs_2_NaBiCl_6_:Yb^3+^/Er^3+^ and Cs_2_Na_0.8_Ag_0.2_BiCl_6_:Yb^3+^/Er^3+^ (2×2×1 supercells) as the examples (Figure S10, Supporting Information), providing insights into the variation underlying luminescence mechanisms after alloying of Ag^+^ and Na^+^ using first‐principles density functional theory (DFT) calculations. Among them, the conduction band minimum (CBM) and valence band maximum (VBM) of Cs_2_NaBiCl_6_:Yb^3+^/Er^3+^ are mainly contributed by the Bi‐6p and Cl‐3p states, while the contributions of Na^+^, Yb^3+^, and Er^3+^ to VBM and CBM are negligible (**Figure** **4**a). On the contrary, the VBM of Cs_2_Na_0.8_Ag_0.2_BiCl_6_:Yb^3+^/Er^3+^ was mainly contributed by the Ag‐4d and Cl‐3p states (Figure 4b), which stemmed from the fact that the strongly covalent Ag─Cl bond connected rapidly the neighboring octahedra after Ag^+^ doping, thereby breaking the ionization state between Na^+^ and Cl ions, accompanied by the shrinking of the wide bandgap (Figure S11, Supporting Information). Meanwhile, the neighboring [BiCl_6_]^3−^ transforms from a relatively regular octahedron to a distorted form after Ag^+^ and Na^+^ alloying, the local site symmetry of Bi^3+^ in Cs_2_Na_0.8_Ag_0.2_BiCl_6_:Yb^3+^/Er^3+^ is reduced (Figure 4c). Thus, the parity forbidden of band edges in Cs_2_NaBiCl_6_:Yb^3+^/Er^3+^ was broken under the Jahn‐Teller effect, which facilitated the absorption and excitation of Bi^3+^, ensuring its stable DC fluorescence emission.

**Figure 5:**
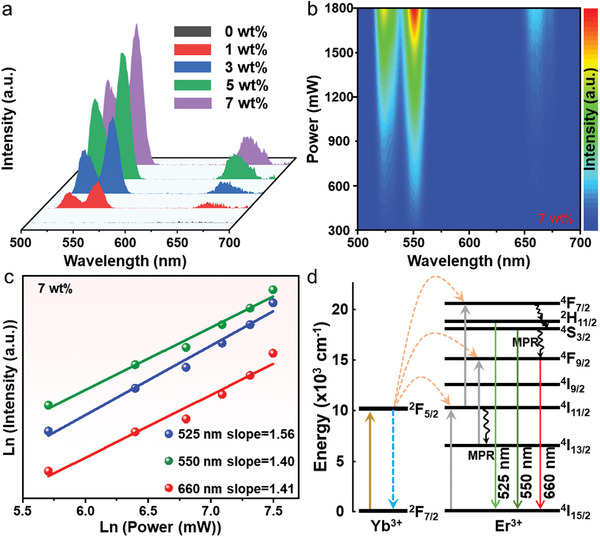
a) UC (980 nm excitation) PL spectra of CFs with varying DP content. b) UC PL spectra of 7 wt.% DP/P(VDF‑TrFE) CFs under 980 nm pumping with different laser powers. c) The Ln‐Ln plots of the UC luminescence intensity versus excitation power. d) UC PL mechanism of Cs_2_Na_0.8_Ag_0.2_BiCl_6_:Yb^3+^/Er^3+^.

Additionally, to confirm the multimodal fluorescence emission of CFs, the up conversion (UC) fluorescence properties of CFs with varying DP content were probed under the excitation of 980 nm IR light. The UC spectra of CFs showed three distinct narrow emission peaks, which are located at 525, 550, and 660 nm (**Figure** **5**a), belonging to the typical multi‐peak luminescence characteristics of rare earth ions, corresponding to the color coordinates at (0.26, 0.71) (Figure S8, Supporting Information). Meanwhile, the UC fluorescence intensity of CFs was significantly enhanced with the elevated DP content, which was consistent with its DC fluorescence change behavior, and the UC PLQY of 7 wt.% DP/P(VDF‐TrFE) CFs with the most outstanding optical performance was 0.57% (Figure S9b, Supporting Information), suggesting that the fluorescence output of CFs was mainly determined by the DP content. Immediately after, the photon transmission process of the three luminescent peaks of Cs_2_Na_0.8_Ag_0.2_BiCl_6_/Yb^3+^, Er^3+^ was simulated by collecting the UC PL spectra of 7 wt.% DP/P(VDF‑TrFE) CFs with the best optical performance under different excitation light powers (Figure 5b). UC luminescence usually involves a process participating multiple photons, and the following relationship exists between the intensity of the emitted visible light and the power of the excitation light,^[^ ^46^, ^47^ ^]^

(1) $$l_{\mathrm{uc}}\propto {\left(l_{\mathrm{laser}}\right)}^{n}$$

where I_uc_ is the intensity of the upconverted luminescence, I_laser_ is the laser power used for excitation, and n is the number of photons to be absorbed per photon emitted. The fitting results indicated that the photon absorption numbers corresponding to the 525, 550, and 660 nm emission peaks are 1.56, 1.40, and 1.41, respectively (Figure 5c), which suggested that the UC emission of CFs mainly originated from two‐photon absorption. Among them, Yb^3+^ in Cs_2_Na_0.8_Ag_0.2_BiCl_6_:Yb^3+^/Er^3+^ had a relatively larger IR absorption cross‐section at 980 nm, and the absorbed energy could be transferred efficiently into Er^3+^, so the UC luminescence of Er^3+^ in DP was mainly achieved by energy transfer (ET). The specific process was described as the transition of Yb^3+^ from the ground state to the excited state by absorbing a 980 nm photon and transferring the energy to Er^3+^ enabling it to be transitioned to the excited state. Subsequently, Yb^3+^ returned to the ground state and continued to absorb energy, and Er^3+^ at the excited state also proceeded to absorb the energy transferred from Yb^3+^, thereby inducing a higher energy level transition. Finally, Er^3+^ returned to the ground state (^4^I_15/2_) by radiative transition, and emitted characteristic green light. By matching the emission peaks with the energy levels of the rare earth ions, it can be concluded that the three emission peaks correspond to the ^2^H_11/2_→^4^I_15/2_ transition, ^2^S_3/2_→^4^I_15/2_ transition and ^4^F_9/2_→^4^I_15/2_ transition of Er^3+^, respectively (Figure 5d). Through the above systematic analysis of the optical properties for CFs, it can display different fluorescence emission under two excitation sources (UV and IR) (Figure S8, Supporting Information), laying a solid foundation for the subsequent application in multi‐modal fluorescence anti‐counterfeiting.

**Figure 6:**
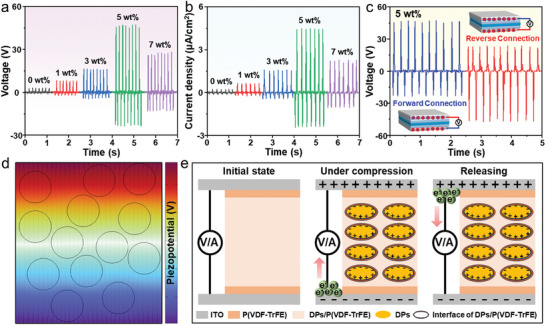
The output a) voltage and b) current density of CFs with varying DP content. c) Piezoelectric output voltage of 5 wt.% DP/P(VDF‑TrFE) CFs in the forward and reverse connection. d) COMSOL simulation of the piezoelectricity generated by external stress. e) The working mechanism of CFs under the whole “press‐release” cycle.

Considering the differences in the piezoelectric properties of CFs with varying DP content, their electrical properties were systematically investigated to ensure optimal performance in piezoelectric anti‐counterfeiting. Since the orientation of DP in the P(VDF‑TrFE) film is random, the power generation properties of CFs were enhanced by polarizing the electric dipoles of DP and P(VDF‑TrFE) before testing (Figure S12, Supporting Information).^[^ ^48^ ^]^ The piezoelectric output of the CFs during compression and release was measured using a linear motor with a contact area of 0.64 cm^2^ and a force of 30 N (0.469 MPa) at 5 Hz frequency. **Figure** **6**a,b demonstrated the dependent variation in the output power of CFs with increased DP content. The open‐circuit voltage (V_oc_) and short‐circuit current density (J_sc_) values of CFs were enhanced when the DP content was increased (Figure S13, Supporting Information), which mainly originated from the fact that DP with an appropriate amount can promote effectively the crystallization of the β‐phase in P(VDF‑TrFE) film. Furthermore, P(VDF‑TrFE) film can play a protective role in reducing the charge dissipation of DP, resulting in a significant improvement of V_oc_ and J_sc_ outputs at low DP content. However, the enriched DP would transfer partially to the surface of the P(VDF‑TrFE) film and be exposed to the environment when the DP content continued increasing, thus affecting the contact between the CFs and the electrodes, causing their output power to be attenuated (Figure S13, Supporting Information), and similar phenomena have been found in other piezoelectric films.^[^ ^38^, ^48^, ^49^ ^]^ Among them, the piezoelectric properties of 5 wt.% DP/P(VDF‑TrFE) CFs are the most prominent, with a maximum V_oc_ output reaching 47.1 V and a J_sc_ output of 4.45 µA cm^−2^, corresponding to a piezoelectric strain constant (*d*_33, eff_) value of 25.3 pC N^−1^ (Figure S13, Supporting Information). The surface potential of 5 wt.% DP/P(VDF‐TrFE) CFs was determined to be 366.2 mV by using Kelvin probe force microscopy (KPFM) and an electrostatic voltmeter, suggesting that DP enhanced P(VDF‑TrFE) film can trap and maintain the induced electrons (Figure S14, Supporting Information). In addition, 5 wt.% DP/P (VDF‐TrFE) cf was obtained by using spin‐coating method, comparing the influence of screen‐printing and spin‐coating process on the optical and electrical properties of CFs (Figure S15, Supporting Information). The results showed that the average crystal size of DP in the CFs obtained by the spin‐coating method was also 900 nm (Figure S15a, Supporting Information), corresponding to a polar β‐phase ratio of 46% and a semipolar phase γ‐phase of 35% (Figure S15b and Table S2, Supporting Information), which was basically similar to that of screen‐printed CFs, suggesting that the electroactivity of the CFs was mainly related to the DP content. Subsequently, the UC and DC PL spectra of the CFs indicated that it also had an excitation wavelength‐dependent (Figure S15c,d, Supporting Information), which corresponded to a maximum V_oc_ output of 46.8 V and a J_sc_ output of 4.42 µA cm^−2^(Figure S15e,f, Supporting Information). Overall, the changed processes did not influence the performance of the CFs, and the spin‐coated and screen‐printed films had similar morphological, optical and electrical properties. At the same time, the power output of the CFs can be freely switched between forward and reverse connections (Figure 6c). Positive output signals are observed when CFs at forward connection, and opposite output signals are measured under reverse connection, whose values of V_oc_ are essentially the same (47.1 V), and the reversible electrical output power confirmed that the power output was derived from the piezoelectric effect of the CFs.^[^ ^50^ ^]^ In addition, the positive piezoelectric output signal was always larger than the negative signal in each “press‐release” cycle with the forward connection (Figure 6c), owing to the difference in strain rates between the applying and releasing stress. The strain rate of CFs was reduced during stress release due to the relaxation of the soft P(VDF‑TrFE) molecular chains, thus lowering the accumulated charge.^[^ ^48^, ^49^ ^]^ Subsequently, finite element simulations based on the COMSOL software package were performed to qualitatively analyze the stress and piezoelectric potential in the piezoelectric CFs. Most of the mechanical stresses are concentrated around the piezoelectric spheres when pressure was applied to the top surface of the composites, suggesting that there was an effective stress‐transfer capability within the CFs (Figure S16, Supporting Information).^[^ ^51^ ^]^ Meanwhile, according to the direct piezoelectric effect, the corresponding piezoelectric potential difference of a piezoelectric material also increased when it was subjected to an enhanced stress. Therefore, benefiting from the piezoelectric composite modal has high stress‐transfer capability, which allows the piezoelectric potential on the upper surface of the CFs to be higher than that on the lower surface (Figure 6d). Finally, the piezoelectric working mechanism of 5 wt.% DP/P(VDF‑TrFE) CFs was analyzed in depth (Figure 6e). During the initial stage, the CFs was not exposed to any external stress, so there was no charge accumulation on the electrode surface. When the CFs are compressed, a piezoelectric charge on the two electrode surfaces will be generated, resulting in a potential difference. Accompanied by external free charges migrating to the electrodes and accumulate to counteract the piezoelectric potential, thereby generating a positive signal. In contrast, when the piezoelectric potential disappears during stress relaxation, the accumulated charge will be reversed, thus collecting a negative electrical signal. In summary, 5 wt.% DP/P(VDF‐TrFE) CFs possess excellent piezoelectric characteristics, which enable their output electrical signals to be quickly collected and recognized by professional equipment, thus realizing re‐encryption and protection of anti‐counterfeiting information.

**Figure 7:**
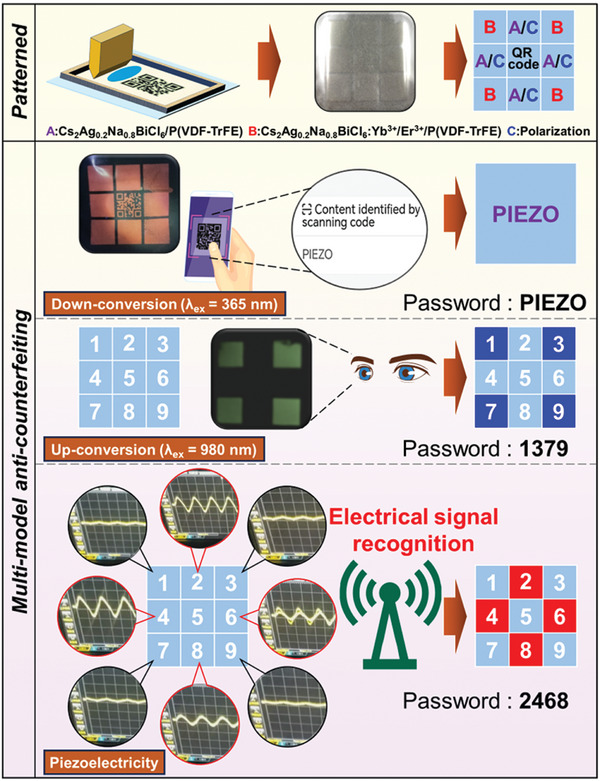
Patterned fluorescent piezoelectric film in advanced anti‐counterfeiting applications.

Combined with the excitation wavelength‐dependent PL modulation properties and piezoelectric response exhibited by CFs with varying DP content, 5 wt.% DP/P(VDF‐TrFE) CFs were preferred for the development of multimodal encryption anti‐counterfeiting applications. Figure S17 (Supporting Information) presents a schematic of the unlocking process for the encrypted information, the first time CFs are excited by 365 nm UV light, the information corresponding to the orange emission was set as the correct password, and only after the information is entered correctly, the system will skip to the next level. Subsequently, the CFs are excited by 980 nm IR light, and the message corresponding to the green emission was set to the correct password, and if it was entered incorrectly, the system would be locked. Finally, the designated locations of the CFs are pressed by an external force, generating an electrical signal that will be recognized by the system and automatically unlocked. Only after the password has been entered correctly all three times, the system can be unlocked, resulting in upgraded information security. Here, CFs with different components were employed to obtain encrypted fluorescent piezoelectric anti‐counterfeiting film by patterned printing technique (**Figure** **7**). Under UV light, the hidden message “PIEZO” was acquired by using a QR code scanner, and skipping to the next level after entering the correct information. Under IR light, the green fluorescent emission corresponds to the digital message “1379”. Finally, pressing the corresponding position of “2468” in the CFs to generate the correct electrical signal that will be recognized by the system, thus completing the unlocking of the message (Video S1, Supporting Information). Additionally, the protection of other information was also accomplished using patterned printing techniques, and it can be seen that this type of encryption can be controlled, allowing the security level to be increased dramatically (Figure S18 and Video S2, Supporting Information). Even though this was just a preliminary model, 5 wt.% DP/P(VDF‐TrFE) CFs‐based fluorescent piezoelectric film displayed tremendous promise for applications in door access control systems and high‐level message encryption.

## 3 Conclusion

In summary, patterned lead‐free DP/P(VDF‐TrFE) CFs were designed to exploit both fluorescence and piezoelectric effects for multimodal anti‐counterfeiting encryption. Benefiting from Ag^+^ doping to break the parity forbidden and co‐doping of rare earth ions to achieve energy transfer, CFs exhibited excitation‐dependent PL behaviors, displaying bright orange and green light under 365 nm UV and 980 nm IR irradiation, respectively. Additionally, the incorporation of DP not only enhanced the charge trapping ability of CFs, but also improved the ratio of polar crystalline phases in P(VDF‐TrFE). By adjusting the DP content in the P(VDF‐TrFE) matrix, the 5 wt.% DP/P(VDF‑TrFE) CFs with the most outstanding piezoelectric properties were obtained, producing output voltages and currents of 47.1 V and 4.45 µA cm^−2^, respectively, and a corresponding *d*_33, eff_ of 25.3 pC N^−1^. The integration of optical properties and electrical response enabled the patterned 5 wt.% DP/P(VDF‑TrFE) CFs to be recognized with multiple hidden pieces of information under different environments, sui` for applications in information anti‐counterfeiting encryption and door access control systems, etc. The anti‐counterfeiting design presented here will open new opportunities for high‐level information security protection, paving the way for the development of more advanced multi‐modal anti‐counterfeiting devices in the future.

## Conflict of Interest

The authors declare no conflict of interest.

## Supporting information



Supporting Information

Supplemental Video 1

Supplemental Video 2

## Data Availability

The data that support the findings of this study are openly available in [01] at [https://doi.org/], reference number [1].
