# Supplementary material for: Patterned Lead‐Free Double Perovskite/Polymer Fluorescent Piezoelectric Composite Films for Advanced Anti‐Counterfeiting
Source: Adv Sci (Weinh). 2024 Nov 11;12(1):2409692. doi: 10.1002/advs.202409692 (PMC11714162; doi:10.1002/advs.202409692)
Supplement: Supplementary file 1 — Supporting Information [file ADVS-12-2409692-s003.docx]

**Supporting Information**

**Patterned Lead-Free Double Perovskite/Polymer Fluorescent Piezoelectric Composite Films for Advanced Anti-Counterfeiting**

Jindou Shi^1^, Zeyu Wang*^2,3^, Nanxiang Jia^1^, Minqiang Wang^1^, Youlong Xu^1^, Xiangming Li^2,3^ and Jinyou Shao*^2,3^

^1^Electronic Materials Research Laboratory, Key Laboratory of the Ministry of Education International Center for Dielectric Research&Shaanxi Engineering Research Center of Advanced Energy Materials and Devices, Xi’an Jiaotong University, Xi’an 710049, China.

^2^Frontier Institute of Science and Technology (FIST)，Xi’an Jiaotong University, Xi’an 710049, China

^3^Micro- and Nano-technology Research Center of State Key Laboratory for Manufacturing Systems Engineering, Xi’an Jiaotong University, Xi’an 710049, China.

Corresponding Author: E-mail: zeyu.wang@xjtu.edu.cn; jyshao@xjtu.edu.cn

**Experimental Section**

**Materials.** The cesium chloride (CsCl, 99.99%), silver chloride (AgCl, 99.5%), sodium chloride (AgCl, 99.9%), bismuth chloride (BiCl_3_, 99.99%), Ytterbium (III) chloride hexahydrate (YbCl_3_•6H_2_O, 99.99 %), Erbium (III) chloride hexahydrate (ErCl_3_•6H_2_O, 99.99 %), N, N-Dimethylformamide (DMF, 99.9%) were purchased from Aladdin. poly(vinylidene fluoride-trifluoroethylene) (P(VDF-TrFE)) purchased from Sigma-Aldrich. Hydrochloric acid (HCl) and ethanol (C_2_H_5_OH) were purchased from Sinopharm Chemical Reagent Co., Ltd. All the reagents were used without further purification.

**Preparation of** **Cs_2_Na_0.8_Ag_0.2_BiCl_6_:Yb^3+^/Er^3+^/P(VDF-TrFE) composite films:** The precursor solution of Cs_2_Na_0.8_Ag_0.2_BiCl_6_:Yb^3+^/Er^3+^/P(VDF-TrFE) with different mass fractions (1 wt%, 3 wt%, 5 wt%, 7 wt%,) was obtained by dissolving CsCl (0.036 mmol, 0.108 mmol, 0.18 mmol, 0.252 mmol), NaCl (0.014 mmol, 0.043 mmol, 0.072 mmol, 0.1 mmol), AgCl (0.004 mmol, 0.012 mmol, 0.02 mmol, 0.028 mmol), BiCl_3_ (0.018 mmol, 0.054 mmol, 0.09 mmol, 0.126 mmol), YbCl_3_•6H_2_O (0.00016 mmol, 0.00048 mmol, 0.0008 mmol, 0.0012 mmol), ErCl_3_•6H_2_O (0.00004 mmol, 0.00012 mmol, 0.0002 mmol, 0.0003 mmol) and P(VDF-TrFE) (1 g) in DMF (10 mL) solution, with continued stirring until complete dissolution. Subsequently, Cs_2_Na_0.8_Ag_0.2_BiCl_6_:Yb^3+^/Er^3+^/P(VDF-TrFE) composite films was prepared by spin-coating the precursor solution onto clean glass. After annealing at 60°C, Cs_2_Na_0.8_Ag_0.2_BiCl_6_:Yb^3+^/Er^3+^/P(VDF-TrFE) composite films was finally formed on the glass substrate.

**Preparation of patterned** **fluorescent piezoelectric Cs_2_Na_0.8_Ag_0.2_BiCl_6_:Yb^3+^/Er^3+^/P(VDF-TrFE) composite films:** Firstly, precursor solution A was obtained by dissolving P(VDF-TrFE) in 10 mL of DMF solution, and precursor solution B was obtained by dissolving a mixture of CsCl, NaCl, AgCl, BiCl_3_, YbCl_3_•6H_2_O and ErCl_3_•6H_2_O in 10 mL of DMF solution. Pure P(VDF-TrFE) film was obtained by spin-coating precursor solution A on a glass substrate, and heating and drying at 60°C. Subsequently, precursor solution B was coated on the P(VDF-TrFE) film using screen-printing technique, and the patterned composite films was obtained by heating and drying at 60°C. Finally, the patterned film was again spin-coated with precursor solution A , and heated and dried at 60°C to obtain a sandwich-structured composite films. The composite films obtained were dried at 135°C for 1.5 h, followed by annealing at 152°C and immediate quenching to 0°C. Finally, ITO was deposited on both sides of the composite films by sputtering using a mask. The Cs_2_Na_0.8_Ag_0.2_BiCl_6_:Yb^3+^/Er^3+^/P(VDF-TrFE) composite films was polarized at 1kv for 2h to enhanced its power generation performance.

**Preparation of 5 wt% Cs_2_Na_0.8_Ag_0.2_BiCl_6_:Yb^3+^/Er^3+^/P(VDF-TrFE) composite films by spin- coated method:** All the preparation processes were consistent with the preparation of patterned composite films, with the slight difference that the intermediate functional layer was applied by a spin- coated process, at a speed of 500 rpm.

**Preparation of Cs_2_Na_0.8_Ag_0.2_BiCl_6_:Yb^3+^/Er^3+^ powders:** 0.252 mmol CsCl, 0.1 mmol NaCl, 0.028 mmol AgCl, 0.126 mmol BiCl_3_, 0.0012 YbCl_3_•6H_2_O and 0.0003 mmol ErCl_3_•6H_2_O were mixed into 10 mL of HCl solution, and stirred continuously for 5 h until the precursor material was completely dissolved. Subsequently, the crude product was collected by centrifugation, and washed three times repeatedly with C_2_H_5_OH. Finally, the obtained product was dried at 60°C for 8 h, and Cs_2_Na_0.8_Ag_0.2_BiCl_6_:Yb^3+^/Er^3+^ powders were obtained.

**Characterization Methods.** The morphology and EDS spectra of films were investigated by field emission scanning electron microscopy (SEM, FEI Quatan FEG 250) equipped with an energy dispersive spectrometer (EDS). A Tecnai F20 microscope operated at 200 keV was used to record the TEM images, which were recorded on the 200-mesh carbon-coated nickel grids. The UC and DC PL spectra and PL quantum yields (PLQYs) were recorded on an Edinburgh Instruments FLS 1000 spectrometer combined with a 450 W Xe lamp as well as power adjustable external 980 nm semiconductor laser. The ultraviolet-visible (UV-Vis) absorption and transmittance spectra were recorded by PE Lambda 950. The X-ray diffraction (XRD) patterns were obtained using the DB-ADVANCE X-ray diffraction analyzer diffractometer. X-ray photoelectron spectroscopy (XPS) spectra were measured by a Thermo Fisher ESCALA670B Xi^+^. The Fourier Transform Infrared (FTIR) spectra were obtained using the PerkinElmer Frontier. The surface roughness of the films was obtained from atomic force microscopy (AFM, INNOVA). The surface potential of LPPS-NFC was measured by KPFM (Park Systems NX10) and Electrostatic Voltmeter (Trek, Model 542A). The piezoelectric coefficient of the samples was measured by a standard static piezoelectric constant measuring equipment (Sinocera YE2730). The output voltage and applied force were measured by a mixed system of oscilloscope (Tektronix MDO3024) and force transducer (SINOCERA CLYD-331).

**Density functional theory (DFT) Calculation Details:** We have employed the first-principles to perform DFT calculations within the generalized gradient approximation (GGA) using the Perdew-Burke-Ernzerhof (PBE) formulation. We have chosen the projected augmented wave (PAW) potentials to describe the ionic cores and take valence electrons into account using a plane wave basis set with a kinetic energy cutoff of 500 eV. The GGA+U method was adopted in our calculations. Partial occupancies of the Kohn−Sham orbitals were allowed using the Gaussian smearing method with a width of 0.05 eV. The electronic energy was considered self-consistent when the energy change was smaller than 10^−5^ eV. A geometry optimization was considered convergent when the energy change was smaller than 0.05 eV Å^−1^. The Brillouin zone integration is performed using 2×2×1 Monkhorst-Pack k-point sampling for a structure. Finally, the adsorption energies (Eads) were calculated as Eads= Ead/sub -Ead -Esub, where Ead/sub, Ead, and Esub are the total energies of the optimized adsorbate/substrate system, the adsorbate in the structure, and the clean substrate, respectively.


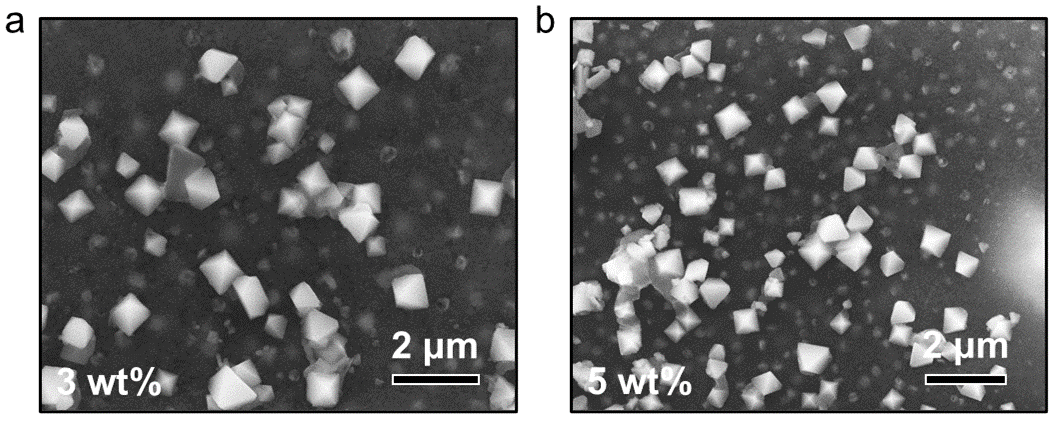


**Figure S1.** SEM images of a) 3 wt% DP/P(VDF‑TrFE) CFs and b) 5 wt% DP/P(VDF‑TrFE) CFs.


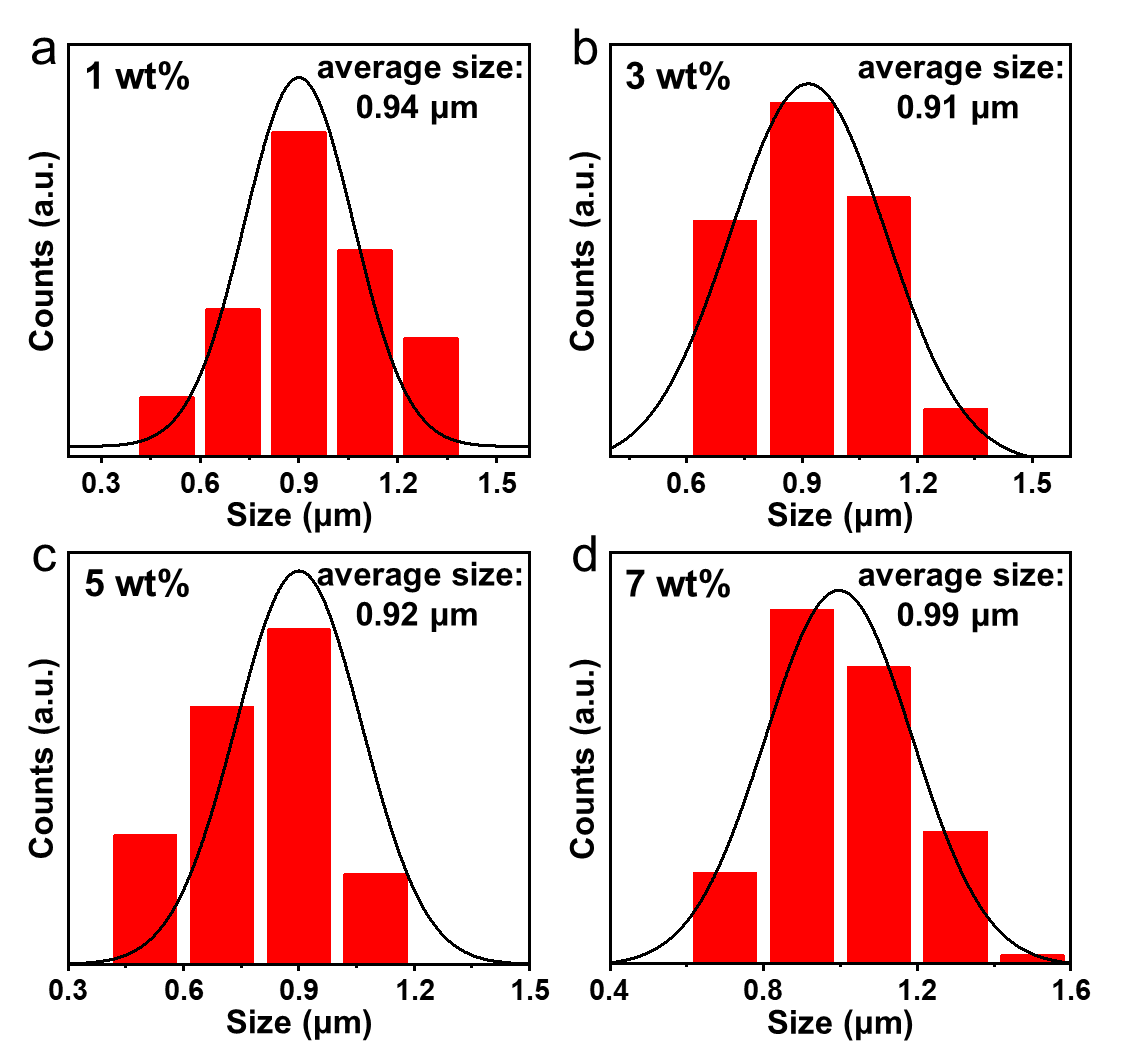


**Figure S2.** The crystal size of the DP in a) 1 wt% DP/P(VDF‑TrFE) CFs, b) 3 wt% DP/P(VDF‑TrFE) CFs, c) 5 wt% DP/P(VDF‑TrFE) CFs and d) 7 wt% DP/P(VDF‑TrFE) CFs.


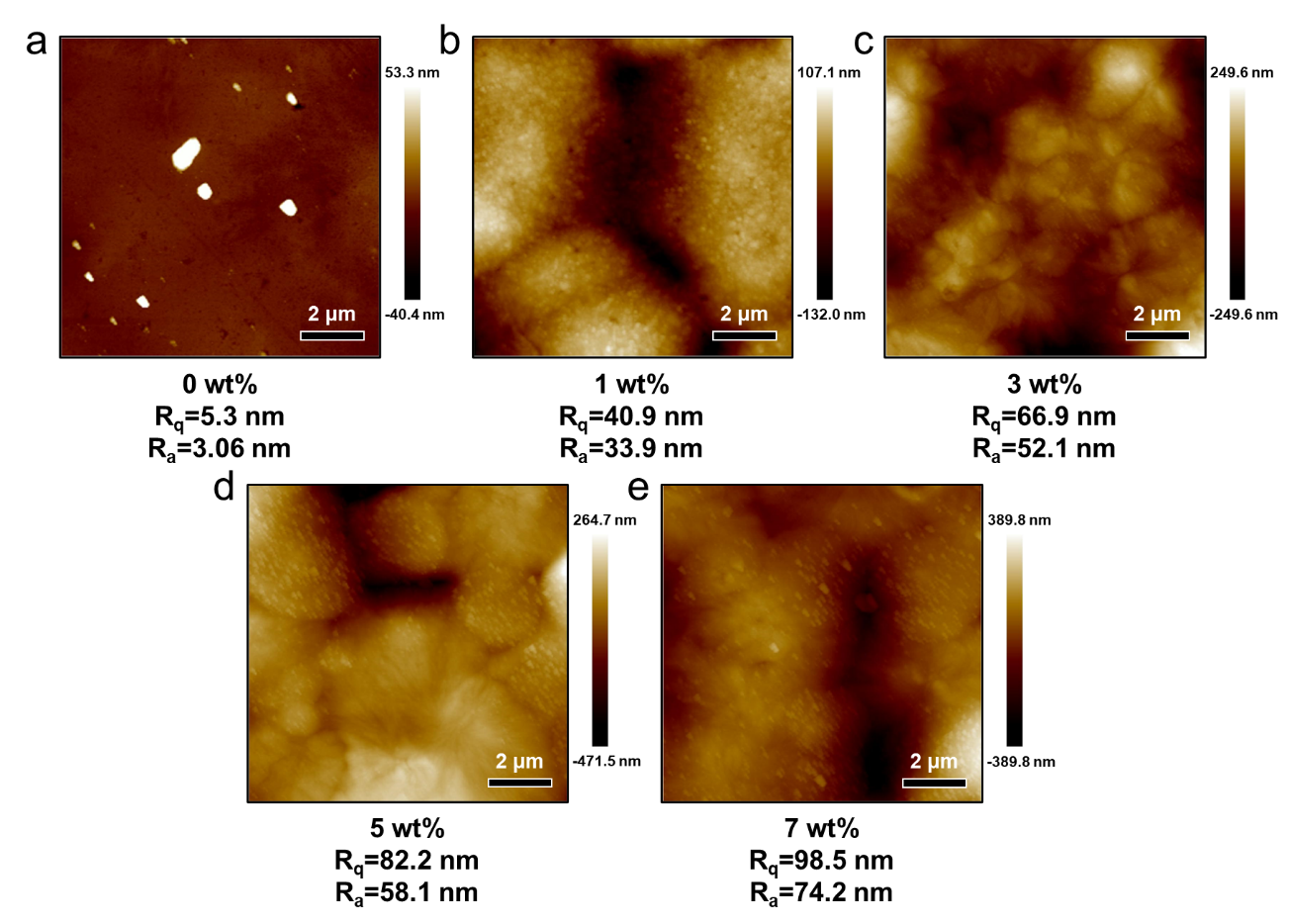


**Figure S3.** AFM images and roughness (a-e) of DP/P(VDF‑TrFE) CFs with different DP content.


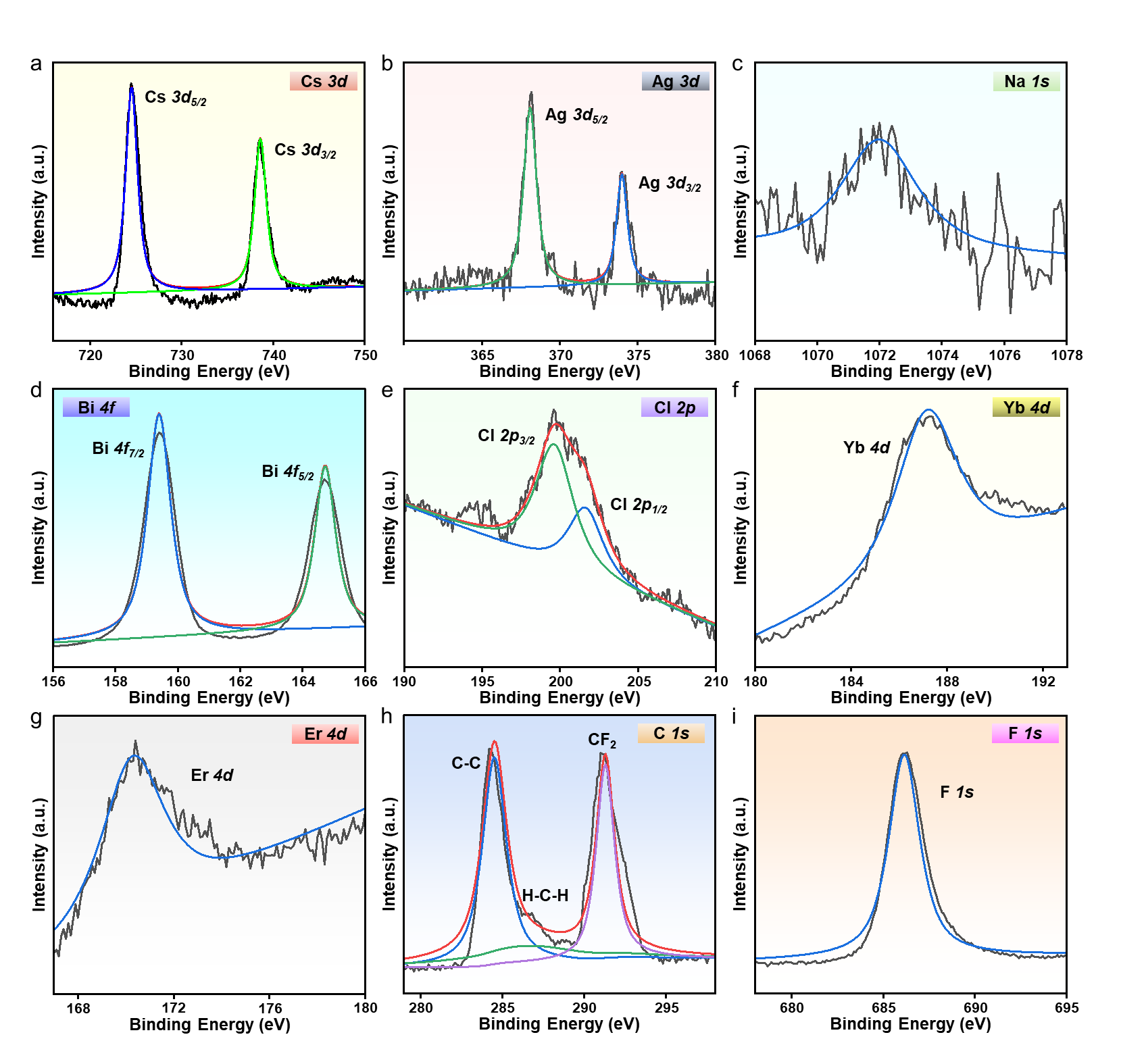


**Figure S4.** High-resolution XPS spectra of all elements in 1 wt% DP/P(VDF‑TrFE) CFs.


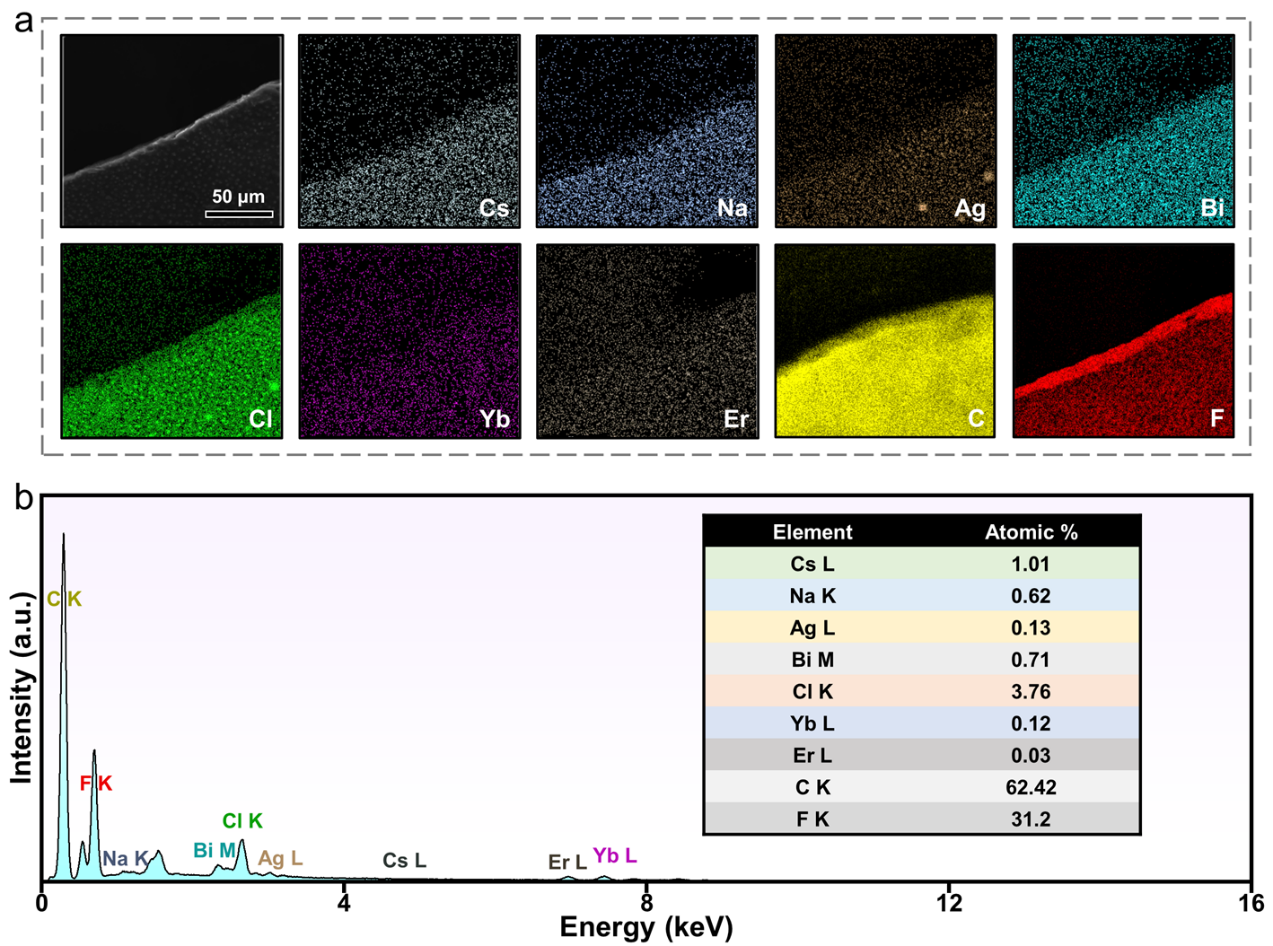


**Figure S5.** a) Mapping of elements in the 1 wt% DP/P(VDF‑TrFE) CFs. b) EDS spectrum of 1 wt% DP/P(VDF‑TrFE) CFs. The inset in b) shows the quant results for the elements in 1 wt% DP/P(VDF‑TrFE) CFs.


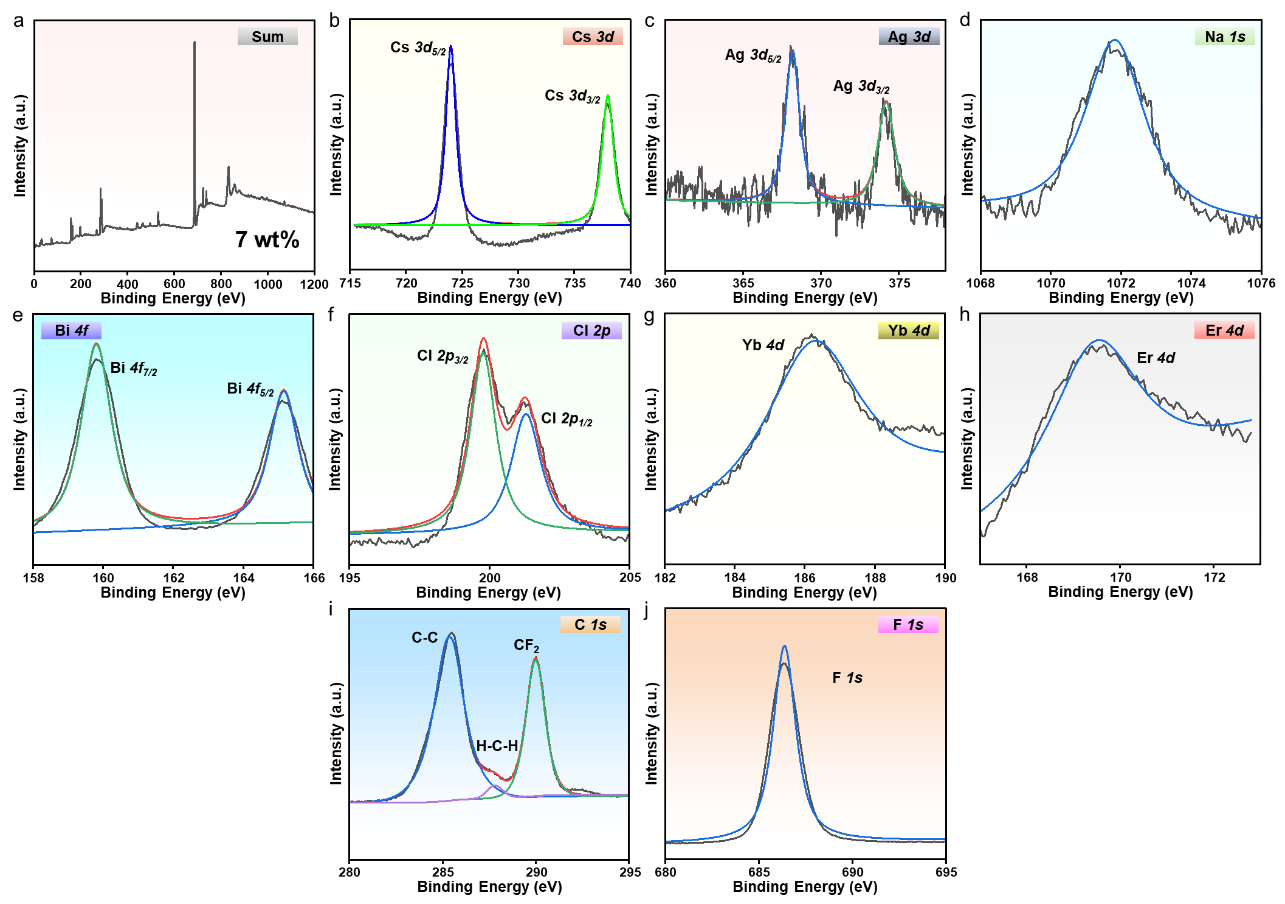


**Figure S6.** a) XPS spectra of 7 wt% DP/P(VDF‑TrFE) CFs. b) High-resolution XPS spectra of all elements in 7 wt% DP/P(VDF‑TrFE) CFs.


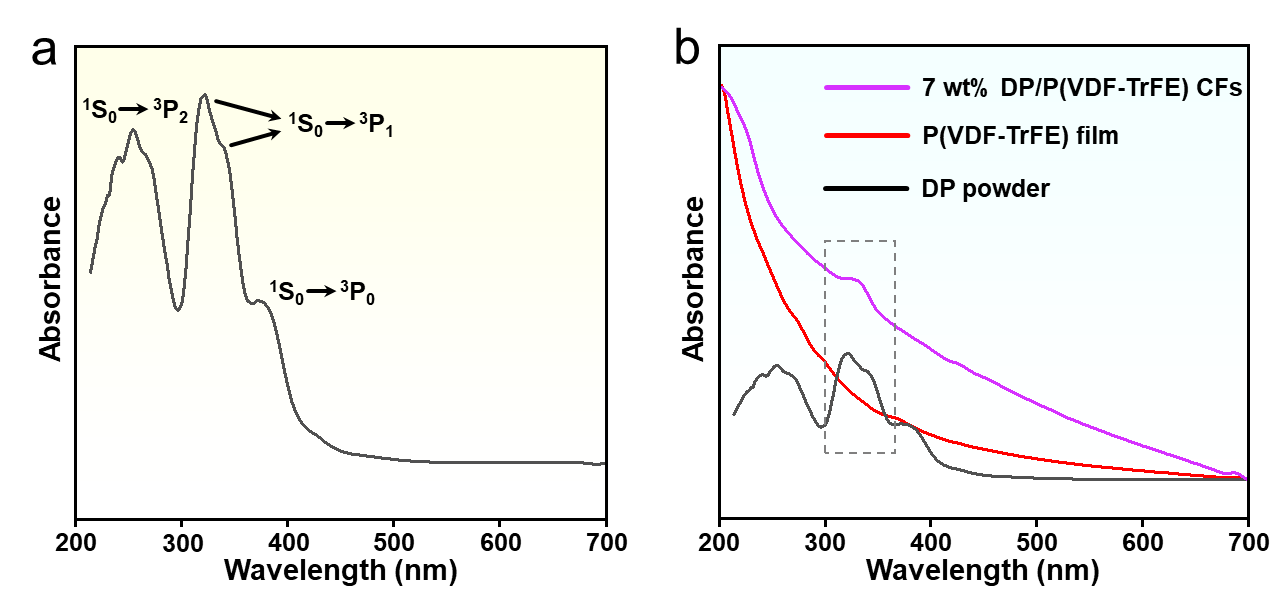


**Figure S7.** Absorbance spectra of a) Cs_2_Na_0.8_Ag_0.2_BiCl_6_:Yb^3+^/Er^3+^ DP powders, b) DP/P(VDF‑TrFE) CFs and P(VDF‑TrFE) film.


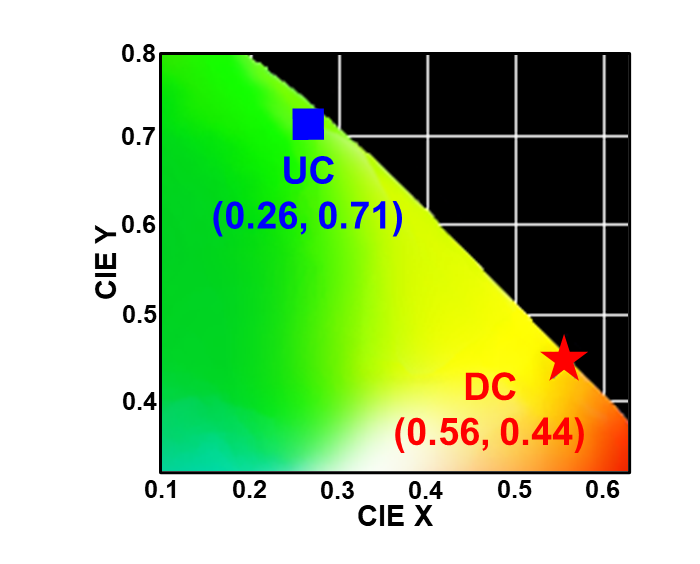


**Figure S8.** The CIE color coordinates corresponding to the DC and UC PL of DP/P(VDF‑TrFE) CFs respectively.


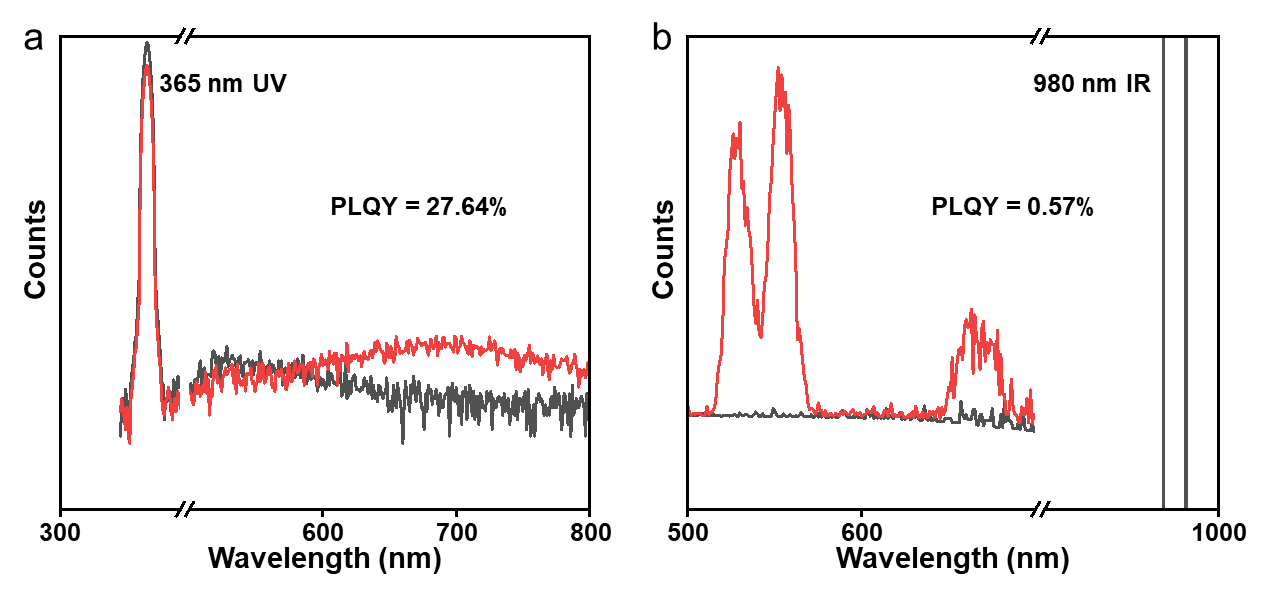


**Figure S9.** The a) DC and b) UC PLQYs of 7 wt% DP/P(VDF-TrFE) CFs.


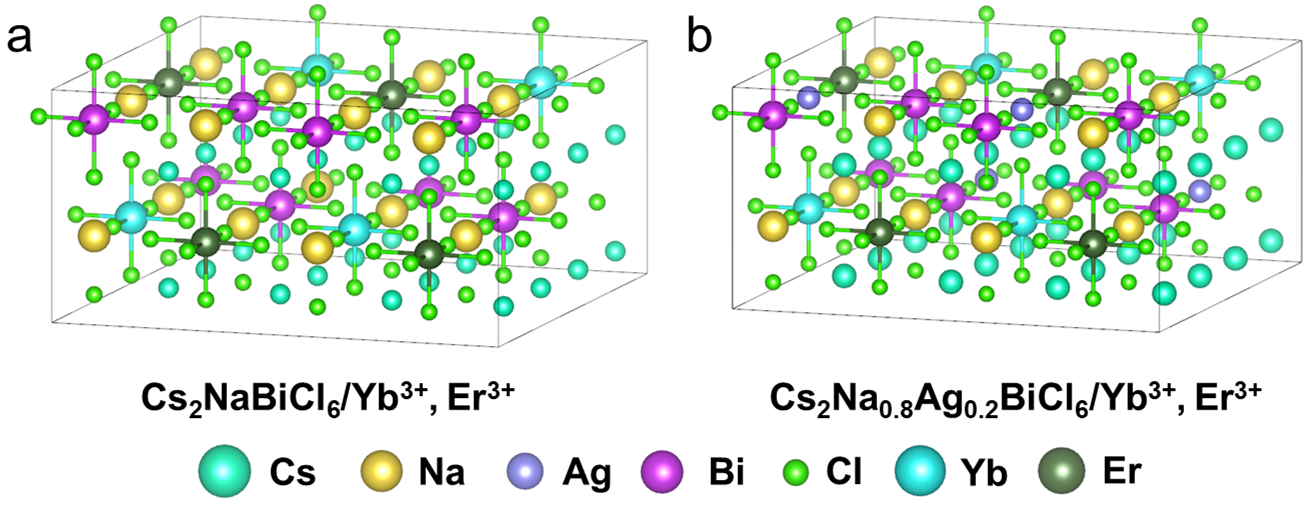


**Figure S10.** Bulk structure models for the a) Cs_2_NaBiCl_6_:Yb^3+^/Er^3+^ and b) Cs_2_Na_0.8_Ag_0.2_BiCl_6_:Yb^3+^/Er^3+^ DPs. These structure models were used for DFT calculations.


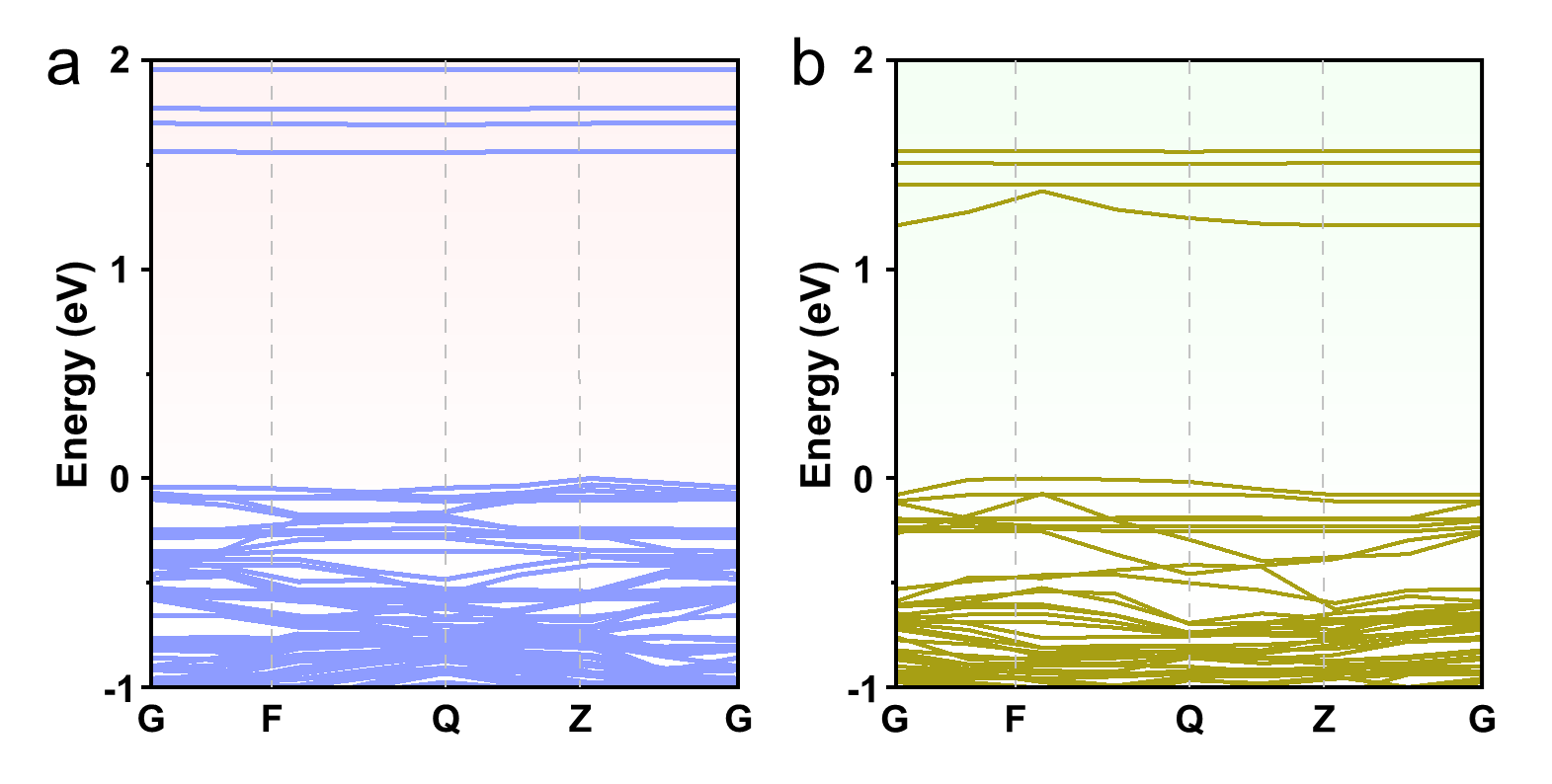


**Figure S11.** Electronic band gap structure of a) Cs_2_NaBiCl_6_:Yb^3+^/Er^3+^ and b) Cs_2_Na_0.8_Ag_0.2_BiCl_6_:Yb^3+^/Er^3+^ DPs.


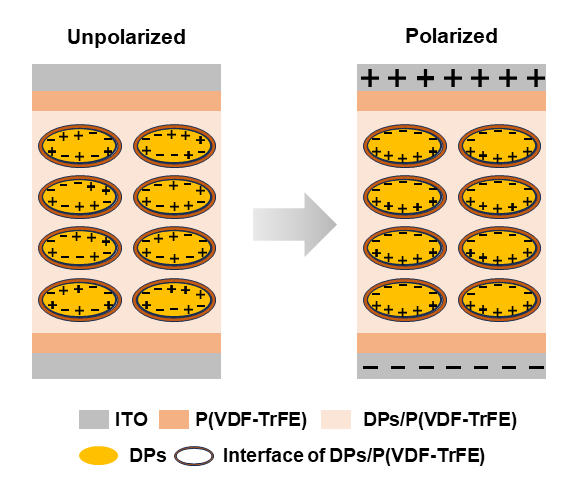


**Figure S12.** a) Working mechanism of the unpolarized CFs. b) Working mechanism of the polarized CFs.


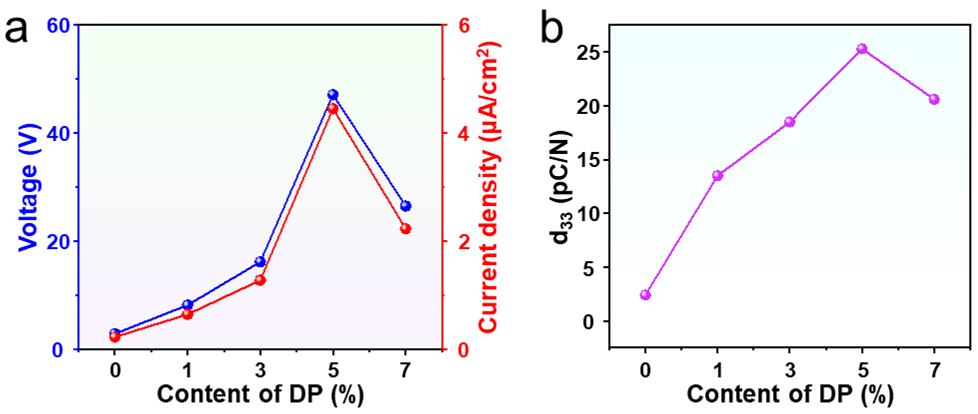


**Figure S13.** The a) output performance and b) piezoelectric strain constant (*d*_33, eff_) of CFs with varying DP content.


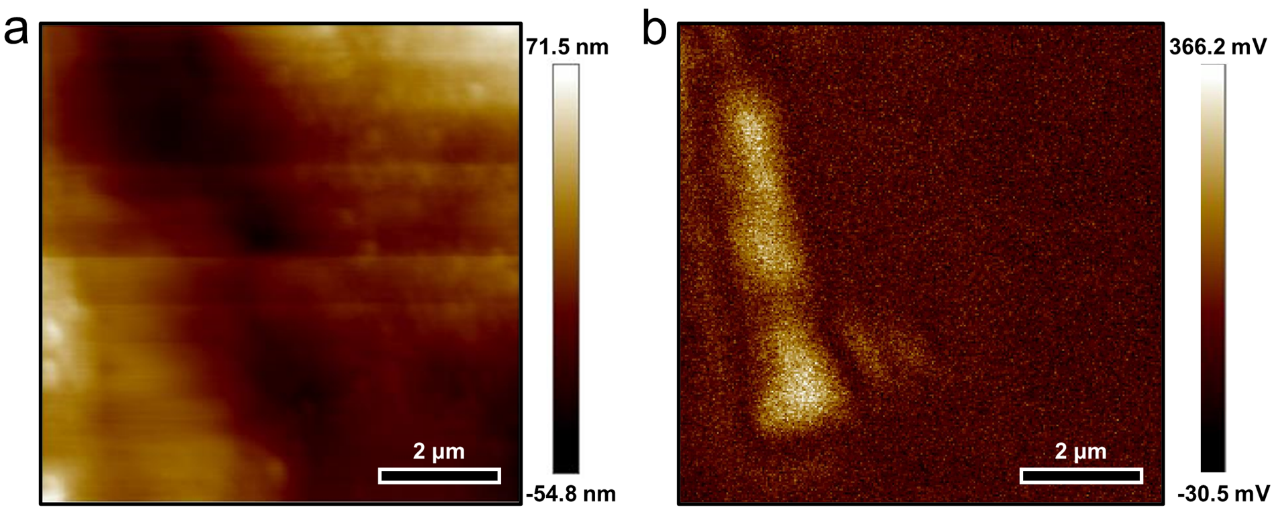


**Figure S14.** Surface potential of 5 wt% DP/P(VDF‑TrFE) CFs.


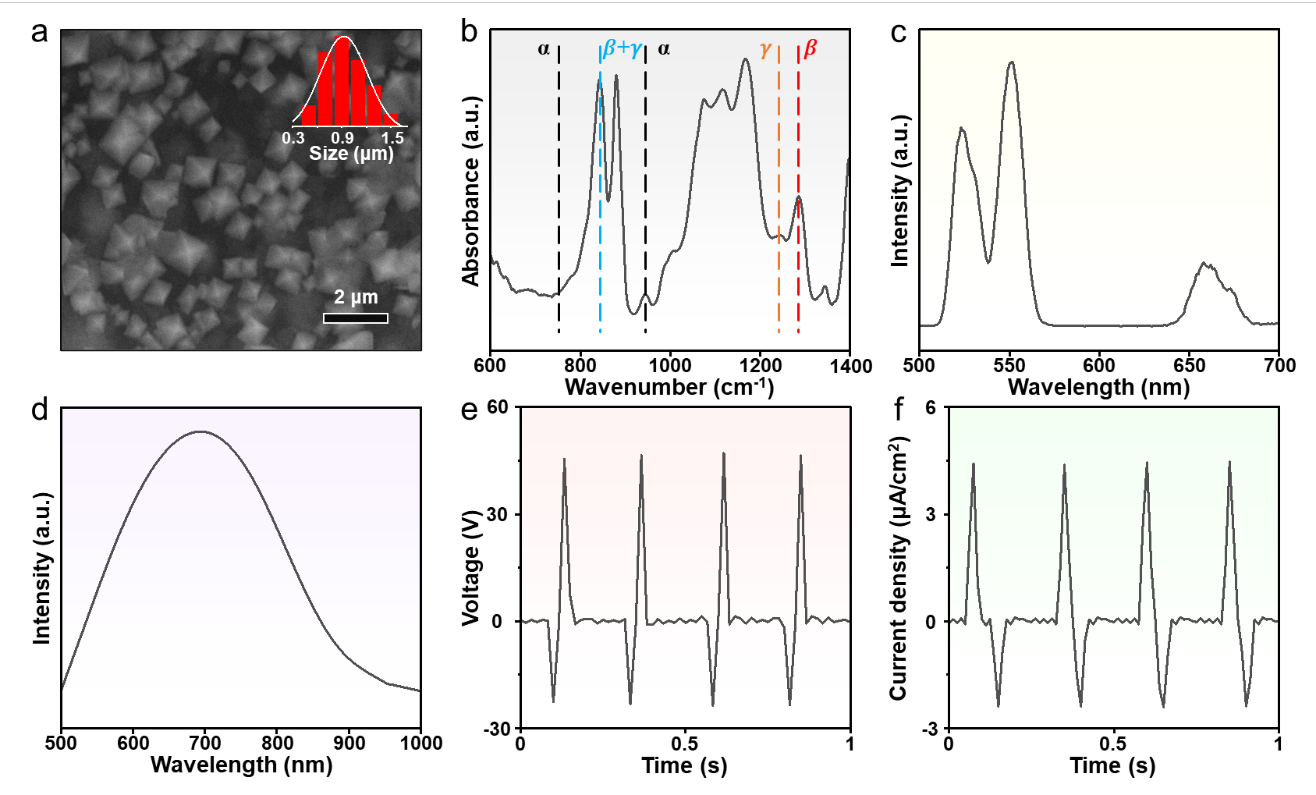


**Figure 15.** a) SEM image, b) FTIR spectrum, c) UC PL spectrum, d) DC PL spectrum, output e) voltage and f) current density of 5 wt% DP/P(VDF-TrFE) CFs was obtained by using spin-coated method.


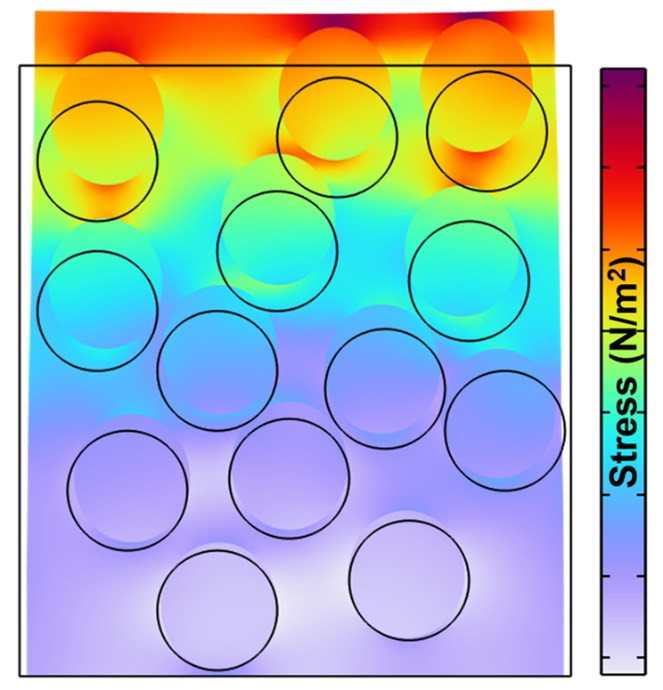


**Figure S16.** COMSOL simulated the stress transfer ability of the model.


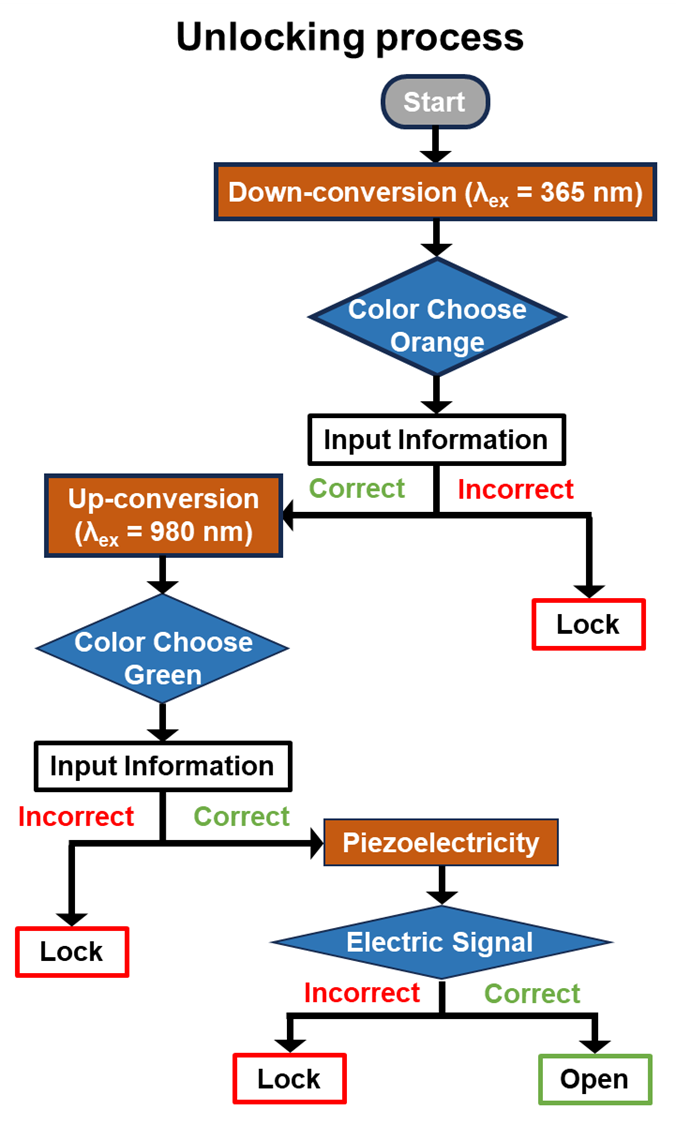


**Figure S17.** Schematic diagram of the unlocking for anti-counterfeiting information.


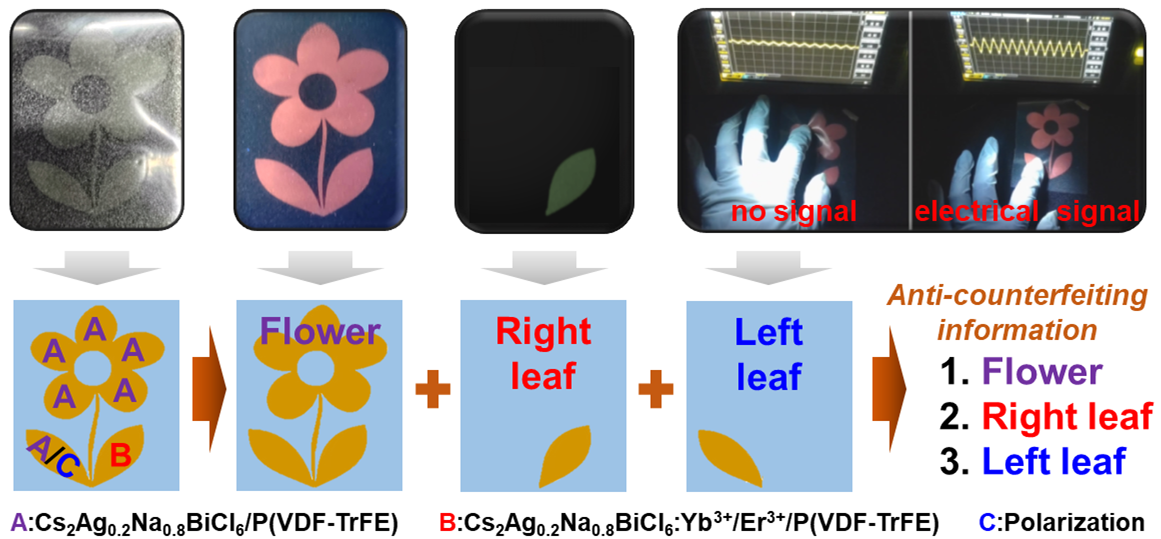


**Figure S18.** Other patterned fluorescent piezoelectric film in advanced anti-counterfeiting applications.

**Table S1.** Electroactive phase of the P(VDF‑TrFE) film with different DP content.

| DP content | α (765 cm^-1^) | β+γ (840 cm^-1^) | β (1275 cm^-1^) | γ (1234 cm^-1^) |
| --- | --- | --- | --- | --- |
| 0 wt% | 59% | 41% | 19% | 22% |
| 1 wt% | 43% | 57% | 28% | 29% |
| 3 wt% | 29% | 71% | 40% | 31% |
| 5 wt% | 17% | 83% | 48% | 35% |
| 7 wt% | 24% | 76% | 44% | 32% |

For a sample containing α-, β- and γ-phase, firstly calculate the content of the polar phases (F(β+γ)) using:

$$F(\beta+\gamma)=\frac{A_{\beta+\gamma}}{(\frac{K_{\beta+\gamma}}{K_{\alpha}})A_{\alpha}+A_{\beta+\gamma}}=\frac{A_{\beta+\gamma}}{1.26\times A_{\alpha}+A_{\beta+\gamma}}$$

where A_α_ and A_β+γ_ are the absorbance at 765 cm^-1^ and 840 cm^-1^, respectively. K_α_ and K_β+γ_ are the corresponding absorbance coefficients, 6.1×10^4^ and 7.7×10^4^ cm^2^ mol^-1^, respectively. F(α) is calculated using 1−F(β+γ). F(β) and F(γ) are calculated using the absorbance of peaks at 1275 cm^-1^ (β-phase) and 1234 cm^-1^ (γ-phase):

$$F\left( \beta\right)=F\left( \beta+\gamma\right)\times\frac{A_{1275}}{A_{1275}+A_{1234}}\times100\%$$

$$F\left( \gamma\right)=F\left( \beta+\gamma\right)\times\frac{A_{1234}}{A_{1275}+A_{1234}}\times100\%$$

**Table S2.** Electroactive phase of 5 wt% DP/P(VDF-TrFE) CFs was obtained by using spin-coated method.

| α (765 cm^-1^) | β+γ (840 cm^-1^) | β (1275 cm^-1^) | γ (1234 cm^-1^) |
| --- | --- | --- | --- |
| 19% | 81% | 46% | 35% |
